# A Pilot Study Examining the Prognostic Utility of Tumor Shrinkage on Cone-Beam Computed Tomography (CBCT) for Stage III Locally Advanced Non-Small Cell Lung Cancer Patients Treated with Definitive Chemoradiation

**DOI:** 10.3390/ijerph18063241

**Published:** 2021-03-21

**Authors:** Kylie H. Kang, Jimmy T. Efird, Tarun K. Podder, Yuxia Zhang, Afshin Dowlati, Mitchell Machtay, Charulata Jindal, Tithi Biswas

**Affiliations:** 1 Department of Radiation Oncology, Washington University School of Medicine and Alvin J. Siteman Comprehensive Cancer Center, St. Louis, MO 63110, USA; kylie@wustl.edu; 2 Cooperative Studies Program Epidemiology Center, HSR&D/DVAHCS, Durham, NC 27705, USA; 3 Department of Radiation Oncology, Case Comprehensive Cancer Center, University Hospitals Cleveland Medical Center, Cleveland, OH 44106, USA; tarun.podder@case.edu (T.K.P.); yuxia.zhang@uhhospitals.org (Y.Z.); axd44@case.edu (A.D.); mxm753@case.edu (M.M.); tithi.biswas@uhhospitals.org (T.B.); 4 Case Western Reserve University School of Medicine, Cleveland, OH 44106, USA; 5 Division of Hematology and Oncology, Department of Medicine, Case Comprehensive Cancer Center, University Hospitals Cleveland Medical Center, Cleveland, OH 44106, USA; 6 Faculty of Science, The University of Newcastle (UoN), Newcastle 2308, Australia; charujindal@gmail.com

**Keywords:** CBCT, imaging, NSCLC, shrinkage, volume

## Abstract

There has been growing interest in utilizing information from cone-beam computed tomography (CBCT) to help guide both treatment delivery and prognosis. In this assessment of locally advanced unresectable stage III non-small cell lung cancer (NSCLC) treated with definitive chemoradiation, we aimed to determine the survival advantage associated with using CBCT to measure tumor regression. Patient, tumor, and treatment characteristics were collected. The serial tumor shrinkage for each patient was determined from tumor volume contours on weekly CBCTs. Survival analysis was performed using the Kaplan–Meier technique and a Cox proportional hazards model. At least two-thirds of patients had a tumor volume reduction of at least 5% after each week of chemoradiation. A weekly reduction in tumor volume of 5% or greater seen on the CBCT images during radiation therapy was significantly associated with improved overall survival, which remained significant when adjusted for age, histology, grade, and T- and N-stages (*p =* 0.0036). Additionally, the presence of N3 disease was associated with a five-fold increased risk of recurrence (*p =* 0.0006) and a nearly three-fold increased risk of death (*p =* 0.053) compared with N0–N2 disease. Tumor volume shrinkage observed in the CBCT images during definitive chemoradiation holds promise as a prognostic indicator of stage III NSCLC, especially given its affordability, availability, and applicability. Further evaluation in a prospective fashion is warranted to validate the tumor volume shrinkage and its clinical utility.

## 1. Introduction

Lung cancer continued to be the leading cause of cancer death among both men and women in 2018, surpassing the number of deaths from colon, breast, and prostate cancers combined [1]. Around 30–40% of patients present with locally advanced non-small cell lung cancer (NSCLC) and are not amenable to surgical resection, so they receive definitive chemoradiation to doses of 60 to 66 Gray (Gy) concurrent with carboplatin/paclitaxel or cisplatin/etoposide [2,3]. However, outcomes continue to remain poor, with a five-year relative survival rate of 19% [1].

One of the challenges of successful radiation treatment is correctly identifying the disease volume while avoiding the surrounding normal tissue. Modern radiation therapy techniques often incorporate image guidance, such as cone-beam computed tomography (CBCT), to optimize planning and dose calculation in order to reduce potential toxicities and improve survival rates [4]. One of the benefits of CBCT is its relatively short acquisition time and utility for monitoring ongoing tumor volume changes during treatment [5,6]. Our study aimed to determine the prognostic potential of tumor regression measured on CBCT images for evaluating survival outcomes in locally advanced, unresectable NSCLC.

## 2. Materials and Methods

### 2.1. Patient Selection

We queried our institutional database to identify patients with stage III NSCLC who underwent definitive radiation therapy with at least 57–66 Gray with chemotherapy. We consecutively identified 42 evaluable stage III NSCLC patients who completed chemoradiation with daily CBCT imaging during external-beam radiation therapy; those without a measurable tumor volume on the CBCT images or lacking sufficient quality CBCT images (preventing uploads into our contouring software) were excluded. Participants were required to have at least one post-treatment follow-up with imaging for disease evaluation and complete treatment details. The tumor, treatment details, and patient characteristics were also collected.

### 2.2. Tumor Regression

For each patient, their daily CBCT images over a six-week period were imported into MIM software (v.6.0; MIM Software Inc., Cleveland, OH, USA). The primary tumor was contoured on every daily CBCT image for the entire treatment duration by a single radiation oncologist to avoid interpersonal variation. To determine the primary tumor regression during radiation therapy, gross tumor volume (GTV) from daily serial CBCT images (for a six-week total) was collected once contoured. Nodal disease was not contoured owing to the limited resolution of the CBCT images. The change in GTV was measured each week per patient, and a variable called “ΔGTV > 5% (T_y_ − T_x_)” was created, defined as the change in GTV greater than 5% between at least two weekly time points (T_y_ and T_x_) during chemoradiation.

### 2.3. Statistical Analysis

All analyses were conducted with SAS, version 9.4 (SAS Institute, Cary, NC, USA). Univariable survival analysis, including recurrence-free survival (RFS) and overall survival (OS), was performed using the product-limit estimator (Kaplan–Meier) technique with the log-rank test. An important advantage of this technique is that all patients do not need to be observed for the entire study period. Survival measures were computed from the date of diagnosis. The OS denoted the maximum time until death or last clinical follow-up if censored, while the RFS also considered the time until a local, regional, or distant failure, whichever occurred first. To determine the influence of patient and disease characteristics on our findings, a multivariable survival analysis, computation of hazard ratios (HRs), and 95% confidence intervals (CIs) were performed using a Cox proportional hazards model. In this model, HRs are defined as the relative instantaneous incidence of mortality between two groups and are used to estimate relative risk. The dependent variable denotes the logarithm of the incidence rate and accounts for the varying lengths of time for follow-ups, while the independent variables are used to model the risk (or hazard) of experiencing an event at a specified point in time, given that one has not experienced the event before that time. The models were tested and confirmed not to deviate from the underlying proportional hazards assumption. For all analyses, a *p <* 0.05 was considered to indicate statistical significance.

## 3. Results

### 3.1. Patient and Treatment Characteristics

The median age was 64 years (range: 44–83), and 69% of patients were female (Table 1). Almost one-third (29%) of patients had N3 disease. Median follow-up was 30 months (range: 2–80). The majority (88%) of patients received a carboplatin-based (versus cisplatin-based) chemotherapy regimen. Three patients received induction chemotherapy, and one patient had sequential (not concurrent) radiation followed by chemotherapy. Almost half (45%) of patients underwent consolidation chemotherapy; those who did not receive it included mostly those with poor performance status or persistent treatment effects (e.g., thrombocytopenia). A median total radiation dose of 60 Gy (range: 50–71.4) was delivered to 28 (67%) patients in 30 fractions (range: 20–33).

### 3.2. Volumetric Tumor Response and Survival Analysis

**Figure 1:**
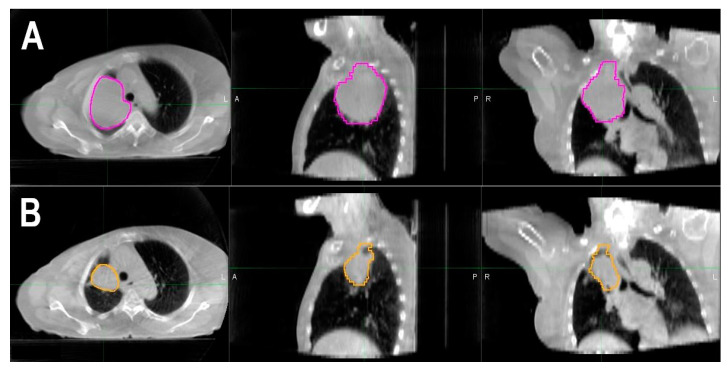
Example of gross tumor volume (GTV) contouring to determine tumor regression during chemoradiation (left to right: axial, sagittal, and coronal views). (**A**) Cone-beam computed tomography (CBCT) of a patient in week 1 of treatment, with GTV contoured in magenta. (**B**) CBCT of the patient in week 6 of treatment, with GTV contoured in gold.

At least two-thirds of patients had a reduction in GTV of at least 5% between each week of chemoradiation (Table 2). Additionally, the GTV decreased in a linear fashion over time during radiation therapy, from a median of 87 cc during week 1 to a median of 51 cc during week 6 (Table 1). Figure 1 is an example of the reduction in GTV seen on one slice of a CBCT image in a patient between weeks 1 and 6 of chemoradiation. The presence of any weekly tumor regression of 5% or greater, as represented by the variable “ΔGTV > 5% (T_y_ − T_x_),” was significantly associated with improved OS, which remained significant when adjusted for age, histology, grade, and T- and N-stages (HR = 0.12, 95% CI: 0.03–0.50; *p =* 0.0036; Table 3). However, this tumor regression was not significantly associated with improved RFS (*p =* 0.53).

**Figure 2:**
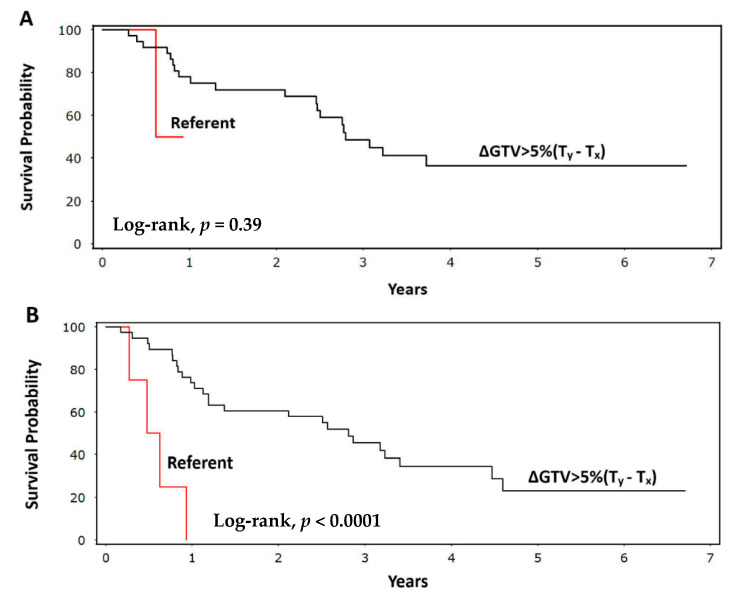
Kaplan–Meier plots showing (**A**) recurrence-free survival (*p =* 0.39) and (**B**) overall survival (*p <* 0.0001) for patients with a change in gross tumor volume (GTV) greater than 5% during at least one week of chemoradiation (ΔGTV > 5% (T_y_ − T_x_)) compared with patients having a change in GTV of less than 5% (referent) during at least one week of treatment.

**Figure 3:**
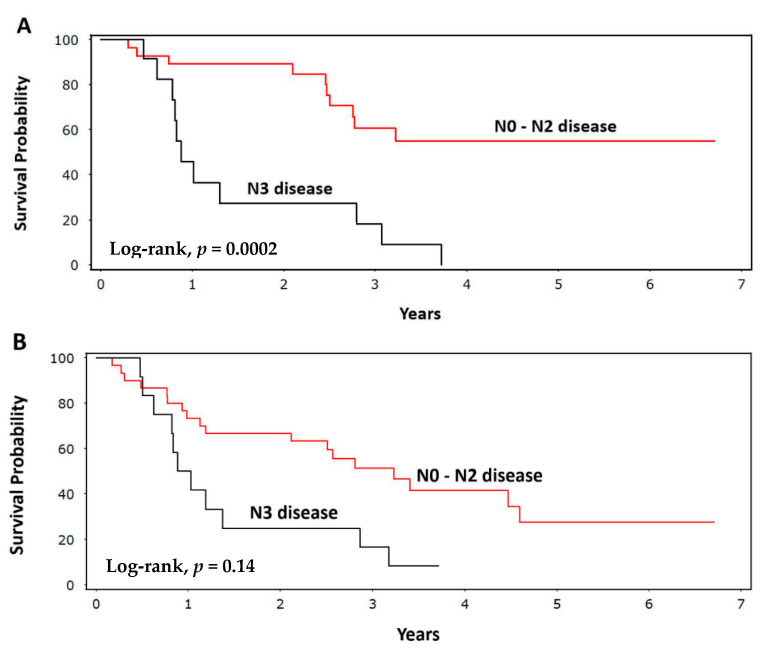
Kaplan–Meier plots showing (**A**) recurrence-free survival (*p =* 0.0002) and (**B**) overall survival (*p =* 0.014) of patients with N0–N2 disease compared with patients having N3 disease.

Consistent with the above findings, the Kaplan–Meier curves illustrated that patients meeting our variable criteria (ΔGTV > 5% (T_y_ − T_x_)) had significantly improved OS (log-rank, *p <* 0.0001), but not improved RFS (log-rank, *p =* 0.39) (Figure 2). The probability of surviving beyond five years among patients with ΔGTV > 5% (T_y_ − T_x_) was 23% (95% CI: 9–41%) versus 0% for the referent group. The presence of N3 disease was associated with worse RFS (log-rank, *p =* 0.0002) and OS (log-rank, *p =* 0.014) compared with N0–N2 disease (Figure 3). This finding remained significant on the multivariable Cox analysis, with N3 disease conferring a five-fold increased risk (HR) of recurrence (95% CI: 2.1–14; *p* = 0.0006) and a nearly three-fold increased risk (HR) of death (95% CI: 1.1–5.8; *p =* 0.053) compared with N0–N2 disease.

## 4. Discussion

Tumor regression, as detected on cone-beam CT (CBCT), may provide essential information that can help to identify a subset of patients who need mid-treatment plan changes and/or closer post-treatment surveillance. This is particularly relevant given that CBCT images are collected routinely as a part of image-guided radiotherapy (RT) and are readily available. Different approaches have been presented in the literature regarding the prognostic utility of information extracted from daily CBCTs [7]. Our study specifically focused on relative tumor volume reduction. We found that a tumor volume reduction of at least 5% as seen on weekly CBCT images during definitive radiation therapy was significantly associated with improved OS. This result is in line with other published studies showing a correlation between tumor shrinkage and improved OS [8,9,10]. On the contrary, our study did not show an association between tumor volume reduction and RFS. There are mixed reports in the literature regarding whether there is an association between tumor shrinkage and locoregional control; this discrepancy between studies may be attributed to being unable to capture the nodal disease status as a part of their analyses, which may play an important role in locoregional RFS. Additionally, the significance of tumor shrinkage in improving OS but not RFS may be explained by the effect of salvage chemotherapy (initiated upon recurrence and thus not influencing RFS) in overall survival; tumors that shrink during radiation therapy may be suggestive of greater response to salvage chemotherapy, which may then improve OS. Lastly, our findings reinforced the well-established effect of nodal stage on survival outcomes in lung cancer [11,12,13,14].

A major strength of our study is the clinical relevance, feasibility, and cost-effectiveness of our prognostic indicator. Moreover, our study is unique from other studies assessing lung tumor shrinkage on CT imaging in that it examined the tumor volume change between weekly treatments rather than throughout the overall treatment period, thereby providing a way to gauge treatment response and a possible prognosis during active treatment. Furthermore, our study exclusively included only stage III NSCLC, which allowed for a more homogenous study population. Having one physician performing all the contouring limits the interpersonal variation in contouring. Our study has limitations, including the exploratory and retrospective nature of our analysis and the small sample size. While the reported effect sizes were statistically significant, a larger sample size would have helped to mitigate the potential influence of residual confounding on the adjusted estimates. In addition, the different chemotherapy timings (induction, sequential, and concurrent) could have biased the tumor shrinkage observed in our study. For instance, concurrent chemotherapy has a known radio sensitization effect with a probability of greater shrinkage compared with induction or subsequent chemotherapy. From studies like the Intergroup (RTOG 9309) trial, it is well known that the pathological complete response of N2 disease is an important prognostic factor in stage III NSCLC [15]. Unfortunately, given the poor resolution of the CBCT images, the nodes could not be evaluated and included in our analysis. The analysis was also not adjusted for multiplicity. Lastly, our findings were limited by the inherent variability between scans, especially for smaller tumor volumes, which may make accurately discriminating weekly volumetric change difficult.

### Clinical Practice Points

There has been growing interest in utilizing information from cone-beam computed tomography (CBCT) to help guide treatment delivery and prognosis of lung cancer patients at risk of poor outcomes.

Survival outcomes were assessed in patients with stage III non–small-cell lung cancer (NSCLC) undergoing definitive chemoradiation to investigate whether a weekly change in tumor volume as measured on daily serial CBCT images by contouring software (MIM) was prognostically useful.

This study found that a weekly reduction in tumor volume of 5% or greater observed in the CBCT images during radiation therapy was significantly associated with improved overall survival, which remained significant when adjusted for age, histology, grade, and T- and N-stages (*p =* 0.0036). This study uniquely assessed the tumor volume change between weekly treatments rather than throughout the overall treatment period, thereby providing a way to gauge treatment response and a possible prognosis during active treatment.

Additionally, the presence of N3 disease was associated with a five-fold increased risk of recurrence (*p =* 0.0006) and a nearly three-fold increased risk of death (*p =* 0.053) compared with N0–N2 disease, which reinforces the well-established effect of nodal stage on survival outcomes in NSCLC.

Tumor volume shrinkage observed in the CBCT images during definitive chemoradiation holds promise as a prognostic indicator of stage III NSCLC. Nonetheless, it is important to reiterate to the reader that the current pilot analysis is not definitive, but rather exploratory in nature. The underlying aims were to generate a hypothesis and to provide the layout and foundational elements for future research on the topic. As the quality of daily CBCT images improves, the progressive shrinkage or lack thereof can provide important information during treatment with the potential for early intervention. New studies will benefit from carefully considering the heterogeneity of response of the tumor tissue to radiation. Ideally, they will also be well-powered to account for type I error and a multiplicity of competing hypotheses.

## 5. Conclusions

In conclusion, the tumor volume reduction observed in the CBCT images during definitive chemoradiation holds promise as a prognostic indicator of stage III, locally advanced NSCLC, especially given its affordability, availability, and applicability. Further evaluation in a prospective fashion is warranted to validate the tumor volume shrinkage and its clinical utility.

## Figures and Tables

**Table 1:** Patient characteristics and treatment (*N =* 42).

Characteristics	*n* (%) Median [IQR]
Age > 65 years	20 (48)
Female	29 (69)
Adenocarcinoma	25 (60)
Poorly differentiated	29 (69)
T-stage 3/4	27 (64)
N3 disease	12 (29)
GTV (cc)	
Week 1	87 (189)
Week 2	75 (171)
Week 3	63 (128)
Week 4	62 (117)
Week 5	58 (109)
Week 6	51 (87)
ΔGTV > 5% (T_y_ − T_x_) *	38 (90)
Carboplatin-based chemotherapy	37 (88)
Consolidation chemotherapy	18 (43)
RT dose, Gy	60 (2)
RT duration, days	44 (6)
Follow-up time, years	2.5 (2.4)

GTV: gross tumor volume; cc: cubic centimeter; RT: radiotherapy; Gy: Gray; * ΔGTV > 5% (T_y_ − T_x_): a greater than 5% reduction in GTV between at least two time points, where T_y_ and T_x_ are two consecutive weeks during a treatment timeline.

**Table 2:** The number of patients with a reduction of gross tumor volume by at least 5% (ΔGTV > 5%) between each week of chemoradiation (*N =* 42).

ΔGTV > 5%	*n* (%)
T_1_ − T_2_ *	32 (76)
T_2_ − T_3_	28 (67)
T_3_ − T_4_	27 (67)
T_4_ − T_5_	28 (67)
T_5_ − T_6_	29 (69)

* e.g., T_1_ − T_2_ indicates the time between week 1 and 2.

**Table 3:** Univariable and multivariable Cox analysis for recurrence-free and overall survival (*N =* 42).

Characteristics	Hazard Ratio (95% Confidence Interval)			
	Recurrence-Free Survival		Overall Survival	
	Univariable	Multivariable	Univariable	Multivariable
Age > 65 years	1.2 (0.52–3.0)	1.2 (0.38–4.0)	1.4 (0.69–3.0)	2.0 (0.80–5.2)
Female	1.2 (0.45–3.4)	1.0 (0.17–5.8)	0.59 (0.28–1.3)	0.28 (0.07–1.1)
Adenocarcinoma	0.61 (0.25–1.5)	0.55 (0.17–1.7)	0.58 (0.27–1.2)	0.82 (0.34–2.0)
Poorly differentiated	1.5 (0.58–4.0)	1.3 (0.27–6.3)	1.1 (0.50–2.4)	2.7 (0.68–11)
T-stage 3 or 4	1.4 (0.53–3.6)	1.7 (0.55–4.9)	1.4 (0.62–3.2)	2.6 (0.99–6.6)
N3 disease	4.6 (1.9–11)	5.4 (2.1–14)	2.6 (1.2–5.6)	2.5 (1.1–5.8)
ΔGTV > 5% (T_y_ − T_x_) *	0.41 (0.05–3.3)	0.46 (0.04–5.2)	0.12 (0.04–0.40)	0.12 (0.03–0.50)

* ΔGTV > 5% (T_y_ − T_x_): a greater than 5% reduction in GTV between at least two time points, where T_y_ and T_x_ are two consecutive weeks during a treatment timeline.

## Disclaimer

The views and opinions expressed in this manuscript are those of the authors. The content does not represent the views of the U.S. Department of Veterans Affairs or the United States Government.
